# Hazardous alcohol consumption and problem drinking in Norwegian and Russian women and men: The Tromsø Study 2015–2016 and the Know Your Heart study 2015–2018

**DOI:** 10.1177/14034948211063656

**Published:** 2021-12-30

**Authors:** Laila A. Hopstock, Alexander V. Kudryavtsev, Sofia Malyutina, Sarah Cook

**Affiliations:** 1 UiT The Arctic University of Norway, Norway; 2 Northern State Medical University, Russian Federation; 3 Novosibirsk State Medical University, Russian Federation; 4 Institute of Internal and Preventive Medicine, Russian Federation; 5 London School of Hygiene & Tropical Medicine, UK; 6 Imperial College London, UK

**Keywords:** Alcohol drinking, alcohol consumption, alcohol drinking habits, epidemiology, cross-sectional studies

## Abstract

**Aim:**

Harmful use of alcohol is a worldwide public health concern. Cultural differences may affect responses to questions on alcohol problems, making international comparisons difficult. We aimed to compare self-reported alcohol consumption and problem drinking between Norwegian and Russian populations.

**Methods:**

We used data from women and men aged 40–69 years participating in the Tromsø Study seventh survey (Tromsø7, *N*=17646, participation 65%), Tromsø (2015–2016), Norway, and the Know Your Heart study (KYH, *N*=4099, participation 51%), Arkhangelsk and Novosibirsk (2015–2018), Russia. Alcohol consumption and problem drinking were measured by the Alcohol Use Disorders Identification Test (AUDIT) via questionnaires (Tromsø7) and interviews (KYH). We compared AUDIT scores and components between populations, by sex.

**Results:**

Non-drinking was more commonly reported in KYH compared with Tromsø7 (men 15.5% versus 4.9%, women 13.3% versus 7.3%). In men, hazardous consumption (41.4% versus 31.5%) and problem drinking (24.8% versus 19.6%) was higher in KYH compared with Tromsø7, but opposite for women (6.5% versus 12.0%, and 2.3% versus 5.8%). KYH men were less likely to report problem drinking behaviours than Tromsø7 men, with the exception of needing a drink first thing in the morning (13.2% versus 2.4%). KYH women consistently reported less consumption and problem drinking than Tromsø7 women.

**Conclusions:**

**We found between-study differences in hazardous drinking, but in men these were lower than suggested by differences in country-level statistics on alcohol consumption and alcohol-related health-harms. Study sample selection, stronger social desirability bias effects in the Russian samples, and cultural differences in responding could have affected the results.**

## Background

Harmful use of alcohol is a major public health problem worldwide [1]. Norway and Russia are neighbouring countries with very different per capita alcohol consumption and policies around availability and cost of alcohol [2, 3]. In Russia, harmful use of alcohol, particularly in men, has been a contributor of premature mortality [4–7]; however, the observed increase in life expectancy during the last decade is associated with a substantial decline in acute alcohol poisoning [8]. Within Norway, the yearly number of alcohol-related deaths (of which acute alcohol poisoning is a minor fraction) has also decreased during this time period [9].

The Alcohol Use Disorders Identification Test (AUDIT) is an established and well-validated internationally used tool for screening for harmful or hazardous alcohol use [10]. While originally designed for use in primary care settings [11] it has been used extensively in epidemiological surveys worldwide [12, 13]. It is designed to measure three domains of alcohol use: alcohol consumption, alcohol dependence and alcohol-related harm, but data from several populations, including Russia [14, 15], supports in reality a two-factor structure: alcohol consumption and alcohol-related problems [16–18]. Two recent papers from a study conducted in acute medically ill patients at two hospitals in Oslo, Norway, and Moscow, Russia, found that self-reported alcohol consumption measured by AUDIT correlates well with the alcohol biomarker Phosphatidylethanol (PEth) in blood [19], but also was overall more sensitive to revealing harmful alcohol use than the biomarker [20].

Amount of alcohol consumed, beliefs and attitudes to drinking, and perceptions of problem drinking are strongly influenced by culture [21–24]. Although the AUDIT is a validated tool, in Russia it has been validated for use only in clinical populations [15, 23], not the general population. Previous studies in Russia have shown that the consumption question on number of drinks included in AUDIT may work less well due to cultural understanding of the concept of a standard ‘drink’ [14, 23]. A recent World Health Organization (WHO) project focused on validation of the AUDIT in Russia [23] additionally identified that the term ‘single drinking occasion’ might not be well understood as a concept in Russia, particularly in a specific group of heavy drinkers who may drink continuously over a period of days.

We hypothesized that differences in cultural beliefs about alcohol and the concept of problem drinking may affect how people answer questions about alcohol problems, leading to conceptual difficulties when making international comparisons. To investigate this we compared how responses to AUDIT questions differed between women and men taking part in population-based surveys within two neighbouring countries with very different drinking cultures – Norway and Russia – and how these differed by demographic factors (age, education and marital status) in each setting.

### Aims

In this study, our aim is to compare self-reported alcohol consumption and problem drinking in women and men in two population-based studies from Norway and Russia, including demographic differences.

## Method

### Sample and data collection

We used data from women and men aged 40–69 years participating in two population-based studies conducted in Norway and Russia in the same time period [25].

The Norwegian sample included participants from the seventh survey of the Tromsø Study [26, 27] (Tromsø7) conducted 2015–2016 in the municipality of Tromsø, Northern Norway. In Tromsø7, all Tromsø citizens aged 40 years and older were invited (*N*=32,591), 21,083 participated (65%) and 17,646 were aged 40–69 years, thus were included in the current analysis. The invitation letter was sent by mail including a study information brochure, a short four-page paper questionnaire, log-in details to complete questionnaires online, and a suggested time for clinical examinations and biological sampling at the study site, which could also be a drop-in. Completion of questionnaires could be performed at home or at attendance, with technical support from research technicians if needed.

The Russian sample included participants from the Know Your Heart (KYH) study [25] conducted 2015–2018 in the two Russian cities Arkhangelsk and Novosibirsk. A random sample of addresses of men and women aged 35–69 from a population list were used to select a random stratified sample within four districts in each city. Recruitment was by home visits from trained interviewers. If participants agreed to take part, a baseline interview was conducted within the participant’s home. They were then invited to attend a health check examination including biological sampling at a polyclinic, where a further interview was conducted by a health care professional. Among those with a valid address where a participant of the correct age and sex was identified, 68% of participants approached in Arkhangelsk and 41% of participants approached in Novosibirsk took part in the baseline interview. Of these, 96% of the participants from Arkhangelsk and 83% from Novosibirsk attended the health check component. In total 4099 participants aged 40–69 years were included in the current analysis.

### Variables

We measured alcohol consumption and alcohol-related problems using AUDIT [11], which is translated to more than 200 languages including Norwegian and Russian. AUDIT consists of 10 questions, of which three are questions on alcohol consumption (frequency and amount, often used as an abbreviated version, AUDIT-C, to calculate alcohol consumption), five are questions on alcohol dependence and two are questions on alcohol-related harm. Minor modifications of questions were used in the Norwegian Tromsø7 version (**Supplementary Figure 1**) and the Russian KYH version (**Supplementary Figure 2**).

In Tromsø7 AUDIT was included as part of the questionnaires (AUDIT-C in the short paper/online questionnaire, the rest of AUDIT in the longer online only questionnaire), where *n*=1149 participants had incomplete AUDIT (missing data on one or more questions). In KYH AUDIT was part of the face-to-face interview conducted at the health check examination where *n*=10 participants had incomplete AUDIT (missing data on one or more questions). Given concerns from previous studies about how the term ‘standard drink’ was interpreted within Russia, a flashcard showing different standard drinks (**Supplementary Figure 3**) was shown to participants in the KYH interview and the interviewers had standardized instructions on how to explain this concept to participants.

We defined hazardous alcohol consumption as AUDIT-C ⩾5 and problem drinking as total AUDIT score ⩾8 in accordance with WHO [10]. Information on education (with or without university education) and marital status (married/cohabitant or single) was collected from questionnaires (Tromsø7) and the baseline interview (KYH).

### Analysis

We compared the prevalence of non-drinking, non-problem drinking and problem drinking by sex and study sample (including strata of site for KYH) (Table I), distribution of characteristics among female (Table II) and male (Table III) non-drinkers, non-problem drinking and problem drinkers, and age-standardized prevalences with 95% confidence intervals (CIs) of hazardous alcohol consumption and problem drinking by sex and study sample (including strata of site for KYH) (Table IV). Further, we compared answers to each AUDIT question by sex and study sample (Table V). Chi-square tests were used to investigate between- (Table I and Table V) and within- (Tables II, III) study differences.

**Table I.** Prevalence of non-drinking, drinking and problem drinking, in women and men, by study and site. The Tromsø Study 2015–2016 and the Know Your Heart study 2015–2018.

	Women				Men			
	Tromsø7	KYH	ARK	NOV	Tromsø7	KYH	ARK	NOV
	(*n*=8606)	(*n*=2379)	(*n*=1255)	(*n*=1144)	(*n*=7819)	(*n*=1729)	(*n*=893)	(*n*=836)
Non-drinking	7.3 (626)	13.3 (317)	11.2 (141)	15.6 (178)	4.9 (386)	15.5 (266)	12.4 (111)	18.8 (157)
Non-problem drinking	86.8 (7471)	84.7 (2014)	86.5 (1085)	82.8 (947)	75.4 (5894)	61.3 (1054)	58.7 (524)	64.2 (537)
Problem drinking	5.9 (509)	2.0 (48)	2.3 (29)	1.7 (19)	19.7 (1539)	23.2 (399)	28.9 (258)	17.0 (142)
*p*-value	<0.001				<0.001			
Total current drinkers	100 (7980)	100 (2062)	100(1098)	100 (964)	100 (7433)	100 (1453)	100 (775)	100 (678)
Problem drinking (current drinkers)	6.4 (509)	2.3 (48)	2.6 (29)	2.0 (19)	20.7 (1539)	27.5 (399)	33.2 (257)	20.9 (142)
*p*-value	<0.001				<0.001			

Numbers are percentages with numbers.

*p*-values are from Chi-square tests for between-study differences (Tromsø7 vs. KYH)

Problem drinking defined as AUDIT score ⩾8.

Tromsø7: the seventh survey of the Tromsø Study 2015–2016; KYH: the Know Your Heart study 2015–2018; ARK: Arkhangelsk; NOV: Novosibirsk; AUDIT: alcohol use disorder identification test.

**Table II.** Study sample characteristics in women by drinking status. The Tromsø Study 2015–2016 and the Know Your Heart study 2015–2018.

		Non-drinker		Non-problem drinker		Problem drinker		Total study population		*p*-value
		Tromsø7	KYH	Tromsø7	KYH	Tromsø7	KYH	Tromsø7	KYH	
		% (*n*)	% (*n*)	% (*n*)	% (*n*)	% (*n*)	% (*n*)	% (*n*)	% (*n*)	
Education	No university education, %	9.2 (339)	15.0 (215)	85.5 (3159)	82.2 (1179)	5.4 (198)	2.7 (38)	100 (3696)	100 (1432)	Tromsø7<0.001 KYH<0.001
	University education, %	5.7 (278)	10.8 (102)	87.9 (4271)	88.2 (835)	6.4 (309)	1.1 (10)	100 (4858)	100 (947)	
Marital Status	Single %	9.1 (178)	15.5 (159)	83.7 (1645)	82.5 (845)	7.3 (143)	2.1 (28)	100 (1966)	100 (1024)	Tromsø 7<0.001 KYH=0.02
	Married/cohabitant, %	6.7 (409)	11.6 (158)	88.0 (5371)	86.3 (1169)	5.3 (323)	2.0 (20)	100 (6103)	100(1355)	
Age	Age 40–49, years	6.5 (205)	8.0 (56)	85.5 (2719)	87.8 (613)	8.0 (255)	4.2 (29)	100(3179)	100 (698)	Tromsø 7<0.001 KYH<0.001
	Age 50–59, years	7.0 (212)	13.6 (107)	87.2 (2625)	84.4 (665)	5.8 (174)	2.0 (16)	100 (3011)	100 (788)	
	Age 60–69, years	8.7 (209)	17.3 (154)	88.0 (2127)	82.4 (736)	3.3 (80)	0.3 (3)	100 (2416)	100 (893)	
	Total	7.3 (626)	13.3 (317)	86.8 (7471)	84.7 (2014)	5.9 (509)	2.0 (48)	100 (8606)	100 (2379)	

Numbers are percentages with numbers.

*p*-values are from Chi-square tests for within-study differences within each drinking status group.

Problem drinking defined as AUDIT score ⩾8.

Tromsø7: the seventh survey of the Tromsø Study 2015–2016; KYH: the Know Your Heart study 2015–2018; AUDIT: alcohol use disorder identification test.

**Table III.** Study sample characteristics in men by drinking status. The Tromsø Study 2015–2016 and the Know Your Heart study 2015–2018.

		Non-drinker		Non-problem drinker		Problem drinker		Total study population		*p*-value
		Tromsø7	KYH	Tromsø7	KYH	Tromsø7	KYH	Tromsø7	KYH	
		% (*n*)	% (*n*)	% (*n*)	% (*n*)	% (*n*)	% (*n*)	% (*n*)	% (*n*)	
Education	No university education, %	5.4 (209)	18.8 (196)	74.3(2871)	56.9 (593)	20.3 (783)	24.4 (254)	100 (3863)	100 (1043)	Tromsø7=0.03 KYH<0.001
	University education, %	4.3 (169)	10.4 (70)	76.5 (2986)	68.2 (461)	19.1 (747)	21.5 (145)	100 (3902)	100 (676)	
Marital Status	Single %	7.5 (102)	18.5 (48)	64.7 (884)	56.4 (146)	27.9 (381)	25.1 (65)	100 (1367)	100 (259)	Tromsø7<0.001 KYH=0.17
	Married/cohabitant, %	4.2 (262)	14.9 (218)	78.2 (4876)	62.2 (908)	17.6 (1097)	22.9 (334)	100 (6235)	100 (1460)	
Age	Age 40–49, years	4.7 (136)	11.8 (56)	73.0 (2111)	56.2 (267)	22.3 (645)	32.0 (152)	100 (2892)	100 (475)	Tromsø7<0.001 KYH<0.001
	Age 50–59, years	5.2 (136)	14.3 (81)	74.4 (1957)	61.7 (349)	20.4 (537)	24.0 (136)	100 (2630)	100 (566)	
	Age 60–69, years	5.0 (114)	19.0 (129)	79.5 (1826)	64.6 (438)	15.5 (357)	16.4 (111)	100 (2297)	100 (678)	
	Total	4.9 (386)	15.5 (266)	75.4 (5894)	61.3 (1054)	19.7 (1539)	23.2 (399)	100 (7819)	100 (1719)	

Numbers are percentages with numbers.

*p*-values are from Chi-square tests for within-study differences within each drinking status group.

Problem drinking defined as AUDIT score ⩾8.

Tromsø7: the seventh survey of the Tromsø Study 2015–2016; KYH: the Know Your Heart study 2015–2018; AUDIT; alcohol use disorder identification test.

**Table IV.** Age-standardized prevalence of hazardous alcohol consumption and problem drinking in women and men by study and site. The Tromsø Study 2015–2016 and the Know Your Heart study 2015–2018.

	Women				Men			
	Tromsø7	KYH	ARK	NOV	Tromsø7	KYH	ARK	NOV
	% (95% CI)	% (95% CI)	% (95% CI)	% (95% CI)	% (95% CI)	% (95% CI)	% (95% CI)	% (95% CI)
Hazardous alcohol consumption (total sample)	12.0 (11.3, 12.6)	6.5 (5.5, 7.6)	6.0 (4.7, 7.5)	7.2 (5.5, 8.8)	31.5 (30.5, 32.5)	41. 4 (39.0, 43.8)	46.8 (43.5, 50.0)	34.8 (31.3, 38.2)
Hazardous alcohol consumption (current drinkers)	12.9 (12.2, 13.6)	7.3 (6.1, 8.5)	6.7 (5.2, 8.1)	8.2 (6.3, 10.0)	33.2 (32.1, 34.2)	48.3 (45.7, 50.9)	52.8 (49.3, 56.3)	42.4 (38.5, 46.3)
Problem drinking (total sample)	5.8 (5.3, 6.3)	2.3 (1.7, 3.0)	2.6 (1.7, 3.6)	2.0 (1.1,2.9)	19.6 (18.8, 20.5)	24.8 (22.7, 26.9)	29.0 (27.1, 33.1)	17.1 (15.3, 21.0)
Problem drinking (current drinkers)	6.3 (5.8, 6.8)	2.5 (1.9, 3.3)	2.9 (1.8, 3.9)	2.2 (1.2, 3.2)	20.7 (19.7, 21.6)	28.8 (26.4, 31.2)	33.9 (30.6, 37.2)	22.0 (18.7, 25.3)

Numbers are age-standardized percentages with numbers or 95% confidence intervals.

Hazardous alcohol consumption defined by AUDIT -C⩾5; Problem drinking defined by AUDIT score ⩾8.

Tromsø7: the seventh survey of the Tromsø Study 2015–2016; KYH: the Know Your Heart study 2015–2018; ARK: Arkhangelsk; NOV: Novosibirsk; AUDIT: alcohol use disorder identification test.

**Table V.** Response to AUDIT items in women and men by study. The Tromsø Study 2015–2016 and the Know Your Heart study 2015–2018.

		Women		Men	
		Tromsø7	KYH	Tromsø7	KYH
		*N* (%)	*N* (%)	*N* (%)	*N* (%)
How often do you have a drink containing alcohol?	Never	691 (7.5)	317 (13.3)	426 (5.1)	266 (15.4)
	Monthly or less	2528 (27.3)	1420 (59.6)	1609 (19.4)	469 (27.2)
	2–4 times per month	3516 (38.0)	578 (24.3)	3435 (41.3)	634 (36.7)
	2–3 times per week	2097 (22.7)	59 (2.5)	2287 (27.5)	271 (15.7)
	4 or more times per week	427 (4.6)	8 (0.3)	558 (6.7)	86 (5.0)
	Total	9259 (100)	2382 (100)	8315 (100)	1726 (199)
	*p-value*	<0.001		<0.001	
How many drinks do you have on a typical day when you are drinking? (Non-drinkers excluded)	1 or 2	5885 (69.3)	1300 (63.0)	3969 (50.6)	421 (28.9)
	3 or 4	2220 (26.1)	569 (27.6)	2656 (33.8)	420 (28.9)
	5 or 6	342 (4.0)	132 (6.4)	866 (11.0)	262 (18.0)
	7,8 or 9	38 (0.5)	46 (2.2)	278 (3.5)	209 (14.4)
	10+	7 (0.1)	17 (0.8)	83 (1.1)	144 (9.9)
	Total	8492 (100)	2064 (100)	7852 (100)	1456 (100)
	*p-value*	<0.001		<0.001	
How often do you have six or more drinks on one occasion? (Non-drinkers excluded)	Never	4921 (57.7)	1624 (78.7)	1805 (23.0)	507 (34.8)
	Less than monthly	3035 (35.6)	372 (18.0)	4353 (55.4)	565 (38.8)
	Monthly	494 (5.6)	49 (2.4)	1313 (16.4)	217 (14.9)
	Weekly	78 (0.9)	18 (0.9)	375 (4.7)	153 (10.5)
	Daily/almost daily	3 (0.03)	0 (0.0)	18 (0.2)	16 (1.1)
	Total	8794 (100)	2063 (100)	7864 (100)	1458 (100)
	*p-value*	<0.001		<0.001	
How often in the last year have you found that you were not able to stop drinking once you started? (Non-drinkers excluded)	Never	7698 (93.2)	2052 (99.4)	6688 (87.2)	1386 (94.9)
	Less than monthly	511 (6.2)	12 (0.6)	850 (11.1)	58 (4.0)
	Monthly	38 (0.5)	0 (0.0)	99 (1.3)	15 (1.0)
	Weekly	11 (0.1)	1 (0.1)	30 (0.4)	0 (0.0)
	Daily/almost daily	3 (0.04)	0 (0.0)	3 (0.04)	1 (0.1)
	Total	8261 (100)	2065 (100)	7670 (100)	1460 (100)
	*p-value*	<0.001		<0.001	
How often in the last year have you failed to do what was normally expected from you because of drinking? (Non-drinkers excluded)	Never	7592 (92.0)	2049 (99.2)	6401 (83.7)	1369 (93.8)
	Less than monthly	641 (7.8)	15 (0.7)	1162 (15.2)	77 (5.3)
	Monthly	13 (0.2)	1 (0.1)	74 (1.0)	9 (0.6)
	Weekly	3 (0.2)	0 (0.0)	12 (0.2)	5 (0.3)
	Daily/almost daily	0 (0.04)	0 (0.0)	2 (0.03)	0 (0.0)
	Total	8249 (100)	2065 (100)	7651 (100)	1460 (100)
	*p-value*	p<0.001		p<0.001	
How often during the last year have you needed a drink first thing in the morning to get yourself going after a heavy drinking session? (Non-drinkers excluded)	Never	8224 (99.6)	2049 (99.3)	7469 (97.6)	1267 (86.8)
	Less than monthly	28 (0.3)	14 (0.7)	144 (1.9)	152 (10.4)
	Monthly	6 (0.1)	2 (0.1)	24 (0.3)	32 (2.2)
	Weekly	0 (0.0)	0 (0.0)	11 (0.1)	6 (0.4)
	Daily/almost daily	0 (0.0)	0 (0.0)	4 (0.1)	2 (0.1)
	Total	8258 (100)	2066 (100)	7652 (100)	1459 (100)
	*p-value*	0.16		p<0.001	
How often during the last year have you had a feeling of guilt or remorse after drinking? (Non-drinkers excluded)	Never	7049 (85.5)	1947 (94.3)	5976 (78.1)	1159 (79.5)
	Less than monthly	1101 (13.4)	105 (5.1)	1500 (19.6)	234 (16.0)
	Monthly	75 (0.9)	12 (0.6)	133 (1.7)	53 (3.6)
	Weekly	16 (0.2)	1 (0.1)	37 (0.5)	8 (0.6)
	Daily/almost daily	5 (0.1)	0 (0.0)	6 (0.1)	5 (0.3)
	Total	8246 (100)	2065 (100)	7652 (100)	1459 (100)
	*p-value*	<0.001		<0.001	
How often during the last year have you been unable to remember what happened the night before because you had been drinking? (Non-drinkers excluded)	Never	7249 (88.0)	2017 (97.7)	5628 (73.8)	1249 (85.6)
	Less than monthly	952 (11.6)	45 (2.1)	1849 (24.2)	180 (12.3)
	Monthly	32 (0.4)	3 (0.2)	138 (1.8)	26 (1.8)
	Weekly	7 (0.1)	0 (0.0)	13 (0.2)	2 (0.1)
	Daily/almost daily	0 (0.0)	0 (0.0)	2 (0.03)	2 (0.1)
	Total	8240 (100)	2065 (100)	7630 (100)	1459 (100)
	*p-value*	<0.001		<0.001	
Have you or someone else been injured as a result of your drinking?	No	8688 (95.5)	2364 (99.3)	7030 (86.2)	1622 (94.0)
	Yes, but not in the last year	388 (4.3)	15 (0.6)	1084 (13.3)	86 (5.0)
	Yes, during the last year	20 (0.2)	3 (0.1)	43 (0.5)	17 (1.0)
	Total	9096 (100)	2382 (100)	8157 (100)	1725 (100)
	*p-value*	<0.001		<0.001	
Has a relative or friend or doctor or another health worker been concerned about your drinking or suggested you cut down?	No	8828 (97.4)	2353 (98.8)	7335 (90.3)	1522 (88.2)
	Yes, but not in the last year	190 (2.1)	8 (0.3)	555 (6.8)	69 (4.0)
	Yes, during the last year	46 (0.5)	21 (0.9)	231 (2.8)	134 (7.8)
	Total	9064 (100)	2382 (100)	8121 (100)	1725 (100)
	*p-value*	<0.001		<0.001	

Numbers are percentages (numbers).

*p*-values are from Chi-square tests.

Tromsø7: the seventh survey of the Tromsø Study 2015–2016; KYH: the Know Your Heart study 2015–2018; AUDIT: alcohol use disorder identification test.

There is missing data for full AUDIT score for Tromsø7 due to one or more questions missing (*n*=1149), and for question-by-question comparison the full set of participants who answered that question is included, therefore denominator is not consistent.

### Ethics and privacy

All participants have given written consent. Tromsø7 was approved by the Regional Committee of Medical and Health Research Ethics (approval 2014/940). KYH was approved by the ethical committees of the London School of Hygiene & Tropical Medicine (approval 8808, 24/02/2015), Novosibirsk State Medical University (approval 75, 21/05/2015), Institute of Preventative Medicine (no approval number, 26/12/2014), and Northern State Medical University (approval 01/01-15, 27/01/2015) and consent for secondary data analysis was given from ethics committees in both sites.

## Results

Non-drinking, drinking and problem drinking in women and men by study and site is shown in Table I. In both women and men, there was a higher proportion of current non-drinkers in KYH (13.3% and 15.5%) than in Tromsø7 (7.3% and 4.9%). Prevalence of problem drinking was higher in KYH men than in Tromsø7 men, both with (27.5% compared with 20.7%) and without (23.2% compared with 19.7%) restriction to current drinkers only, while opposite among women with higher prevalence of problem drinking among Tromsø7 women than KYH women. In KYH, non-drinking was more common and problem drinking less common in women and men in Novosibirsk compared with Arkhangelsk.

Demographic characteristics of non-drinkers, drinkers and problem drinkers in women and men by study are shown in Table II and Table III. The patterns of drinking differed by age, education (except in men in Tromsø 7) and marital status (except in women and men in KYH). Among men the between-study difference in problem drinking decreased with age, with a much larger difference in 40–49-year-olds (32.0% KYH vs. 22.3% Tromsø7) compared with 60–69-year-olds (16.4% KYH vs. 15.5% Tromsø7).

Age-standardized prevalence of hazardous alcohol consumption and problem drinking is shown in Table IV. Hazardous alcohol consumption was higher in KYH men (41.4%) compared with Tromsø7 men (31.5%), while opposite in women with higher hazardous alcohol consumption in Tromsø7 women (12.0%) compared with KYH women (6.5%). Also problem drinking was higher in KYH men (24.8%) than in Tromsø7 men (19.6%), and in Tromsø7 women (5.8%) than in KYH women (2.3%). All study differences were substantively the same when restricted to current drinkers only.

The answers to each AUDIT item for women and men by study are presented in Table V. There were statistically significant differences (*p*<0.001) with all questions on the AUDIT between studies. Tromsø7 women consistently responded in a way indicating a more hazardous drinking pattern than KYH women for every question, for both hazardous consumption and problem drinking. Tromsø7 men were more likely to respond in a manner indicating a more hazardous drinking pattern for the questions on frequency of drinking overall, and all problem drinking behaviours with the exception of needing a drink first thing in the morning to get oneself going after a heavy drinking session (Tromsø7 men 2.4% vs. KYH men 13.2%), and expressions of concern from others about drinking (Tromsø7 men 9.7% vs. KYH men 11.8%). Compared with Tromsø7 men, a higher proportion of KYH men reported non-drinking, but also higher numbers of drinks per occasion and more frequent high alcohol consumption.

## Discussion

In this comparison study of self-reported alcohol consumption and problem drinking in women and men from two population-based studies conducted in Norway and Russia we found that abstaining was more commonly reported in the sample of Russian women and men, compared with Norwegian women and men. At the same time, both hazardous alcohol consumption and problem drinking was more common in the Russian men than the Norwegian men, while the opposite was seen for women with higher prevalence of hazardous alcohol consumption and problem drinking in the Norwegian women compared with the Russian women.

Our findings are consistent with a recent study with comparisons of AUDIT scores from hospital in-patients in Oslo and Moscow [19]. This study also found both higher levels of abstaining and more hazardous drinking consumption among the Russian than Norwegian participants. Our sample is different from the previous study in including participants from population-based studies from different locations in Russia and Norway. These consistencies between findings in different types of study populations and locations strengthen inference about country-level differences in drinking patterns and behaviour.

The WHO global status report on alcohol with data from 2016 [2] reports a per capita alcohol consumption (pure ethanol, recorded and unrecorded combined) in Norway of 3.2 litres in women and 11.6 litres in men, and in Russia 5.8 litres in women and 18.7 litres in men, with Norway showing a relatively stable trend since 2005, and Russia a substantial decrease during the same time period. Further, the report presents alcohol-attributable fractions for deaths from all causes (i.e. percentage of all deaths including both disease and injuries) in Norway of 1.4% in women and 5.4% in men, and in Russia of 19.9% in women and 23.1% in men [2]. Thus, these country-level statistics of alcohol consumption and alcohol-related health-harms are somewhat contradictory to our findings of between-country differences. However, the WHO report presents a non-drinking prevalence in Norway of 30.2% in women and 12.0% in men, and in Russia 44.6% in women and 38.6% in men. Notably, these numbers represent the population aged 15 years and older, but still reflect the same trends as found in our study, i.e. the tendency of dividing into two extremes in the Russian sample particularly in men, with higher levels of both harmful alcohol use and abstaining. Also, our findings may well reflect actual decreasing time trends in alcohol consumption and changes in drinking pattern in Russia observed over the last decade [8]. It is also worth noting the much larger difference in the between-study difference in problem drinking among the younger age group in this study. In both studies the prevalence of alcohol problems decreased with age. This is consistent with findings from most studies worldwide, with harmful alcohol use in general skewed towards a higher impact on the younger population [2].

Cultural beliefs about alcohol and the concept of problem drinking may affect how people answer question about alcohol problems [21–24]. An a priori hypothesis based on existing research was that Russian women and men may underreport the standard number of drinks, suggesting the cultural concept of a standard drink or drinking session might be different [14, 23]. Our findings do not support this hypothesis, as the reason for relatively small difference in AUDIT scores in men between the Russian and the Norwegian sample was not reported number of drinks per occasion, which was higher in the Russian men. It is worth noting that, given previous research from Russia, explicit instructions on how to define a drink were included within the KYH interview, including the use of a pictorial flashcard, which may have mitigated any impact of this. However, we cannot rule out underestimation of the number of drinks, and the actual differences in number of drinks per occasion may actually be larger than those observed in this study. There might also be higher underreporting of alcohol consumption in Russian women. A population survey conducted at both sides of the borders of Karelia in Finland and Russia [28] found evidence of more underreporting of alcohol consumption in Russian compared with Finnish women when comparing AUDIT scores and the level carbohydrate-deficient transferrin and gamma-glutamyltransferase. This is consistent with the recent comparison study of medical patients hospitalized Oslo and Moscow, where the Russian women reported lower alcohol consumption compared with the Norwegian women, but had higher proportion of excessive alcohol use measured by PEth levels, which were suggested to indicate higher degree of underreporting among Russian women due to social desirability [19]. Sex-differences in reporting can partly be explained by between-country differences in gender roles. A previous population study in the city of Novosibirsk using mixed-methods [30] found large sex-differences in drinking patterns, and the qualitative data showed that perceptions about gender roles were a main contributor to the reported drinking behaviour, as women were expected to drink much less than men.

While Russian men reported more hazardous alcohol consumption (defined by AUDIT-C score), Norwegian men were more likely to report most of the specified alcohol problem behaviours than Russian men, with the exception of morning drinking which was more common in Russian compared with Norwegian men. The difference in reporting here is likely to relate to differences in social acceptability of drinking in the morning. In response to concerns about some aspects of the AUDIT in the Russian context, a new Russian-specific version of the AUDIT was recently developed and validated [21] for use in clinical practice. Adaptations focused on clear definitions of a standard drink in AUDIT-C but also adapting the morning drinking question in the full AUDIT to include explicitly reference to hangover drinking (a symptom of dependence) [23], given the higher social acceptability of morning drinking in Russia than in some other countries such as Norway.

While a Russian-specific AUDIT may resolve some of these issues within the clinical context, within the global health research, context standardization of measurements between geographic locations is a key goal in order to make valid comparisons between populations. To date the impact of differences in cultural interpretation of AUDIT questions on findings from comparative studies such as this one is not clear, but our findings support including a detailed breakdown of the AUDIT score components as well as using summary scores when comparing alcohol use between different populations.

### Strengths and limitations

There are several limitations to interpretation of the findings from this study.

First is the difference in data collection methods between the studies. In the Russian study AUDIT was part of a face-to-face interview conducted by a health professional at the examination site, whereas in the Norwegian study AUDIT was part of a self-administered online questionnaire that most participants completed at home before visiting the examination site. Thus, the effect of social desirability bias cannot be ruled out. Another but related factor is cultural differences in answering the questions. An example is the inverse reporting pattern in the sample of Russian men, with fewer symptoms of problem drinking compared with Norwegian men, but not for morning drinking, which has higher social acceptance in Russia but is considered socially unacceptable in Norway. Also, the difference in completeness of AUDIT is likely a reflection of the difference in the data collection method.

Another limitation is the risk of selection bias in both studies. Response proportions were lower for the Russian study, and in particular for the Novosibirsk site. The lower AUDIT scores found in the Novosibirsk study sample than the Arkhangelsk study may indicate higher levels of selection bias in the Novosibirsk study sample. However, they may also reflect genuine geographic variation, given that national statistics from Russia show higher levels of mortality from alcohol poisonings in Arkhangelsk than Novosibirsk. Selection of participants with a healthier lifestyle is a risk in all population-based studies, and both heavy drinkers and abstainers are less likely to re-attend [30], and it is likely that non-attenders in both studies may have a less favorable health profile including a higher prevalence of problem drinking.

Selection bias and social desirability bias are likely to affect all population-based surveys, but because of differences in data collection methods and participation proportions in the two studies, the impact may have been stronger for the Russian sample than the Norwegian sample, leading to an underestimate of the between-study differences. Therefore, the lack of validation of AUDIT categories with biomarkers is an important study limitation.

## Conclusion

Although our study findings should be treated with caution given the limitations described above, we have identified here several differences in drinking patterns and behaviours between participants in population-based studies in Russia and Norway. Further work investigating the impact of drinking culture on responses to AUDIT in different settings and contexts is important to ensure validity in cross-country studies.

## Supplemental Material

sj-docx-1-sjp-10.1177_14034948211063656 – Supplemental material for Hazardous alcohol consumption and problem drinking in Norwegian and Russian women and men: The Tromsø Study 2015–2016 and the Know Your Heart study 2015–2018 Supplemental material, sj-docx-1-sjp-10.1177_14034948211063656 for Hazardous alcohol consumption and problem drinking in Norwegian and Russian women and men: The Tromsø Study 2015–2016 and the Know Your Heart study 2015–2018 by Laila A. Hopstock, Alexander V. Kudryavtsev, Sofia Malyutina and Sarah Cook in Scandinavian Journal of Public Health

sj-docx-2-sjp-10.1177_14034948211063656 – Supplemental material for Hazardous alcohol consumption and problem drinking in Norwegian and Russian women and men: The Tromsø Study 2015–2016 and the Know Your Heart study 2015–2018 Supplemental material, sj-docx-2-sjp-10.1177_14034948211063656 for Hazardous alcohol consumption and problem drinking in Norwegian and Russian women and men: The Tromsø Study 2015–2016 and the Know Your Heart study 2015–2018 by Laila A. Hopstock, Alexander V. Kudryavtsev, Sofia Malyutina and Sarah Cook in Scandinavian Journal of Public Health

sj-docx-3-sjp-10.1177_14034948211063656 – Supplemental material for Hazardous alcohol consumption and problem drinking in Norwegian and Russian women and men: The Tromsø Study 2015–2016 and the Know Your Heart study 2015–2018 Supplemental material, sj-docx-3-sjp-10.1177_14034948211063656 for Hazardous alcohol consumption and problem drinking in Norwegian and Russian women and men: The Tromsø Study 2015–2016 and the Know Your Heart study 2015–2018 by Laila A. Hopstock, Alexander V. Kudryavtsev, Sofia Malyutina and Sarah Cook in Scandinavian Journal of Public Health
