# Supplementary material for: Hazardous alcohol consumption and problem drinking in Norwegian and Russian women and men: The Tromsø Study 2015–2016 and the Know Your Heart study 2015–2018
Source: Scand J Public Health. 2021 Dec 30;51(7):986–94. doi: 10.1177/14034948211063656 (PMC10599088; doi:10.1177/14034948211063656)

**Supplementary Figure 3. Standard drink flashcard (in Russian) used for completion of the AUDIT interview in the Know Your Heart study 2015-2018.**


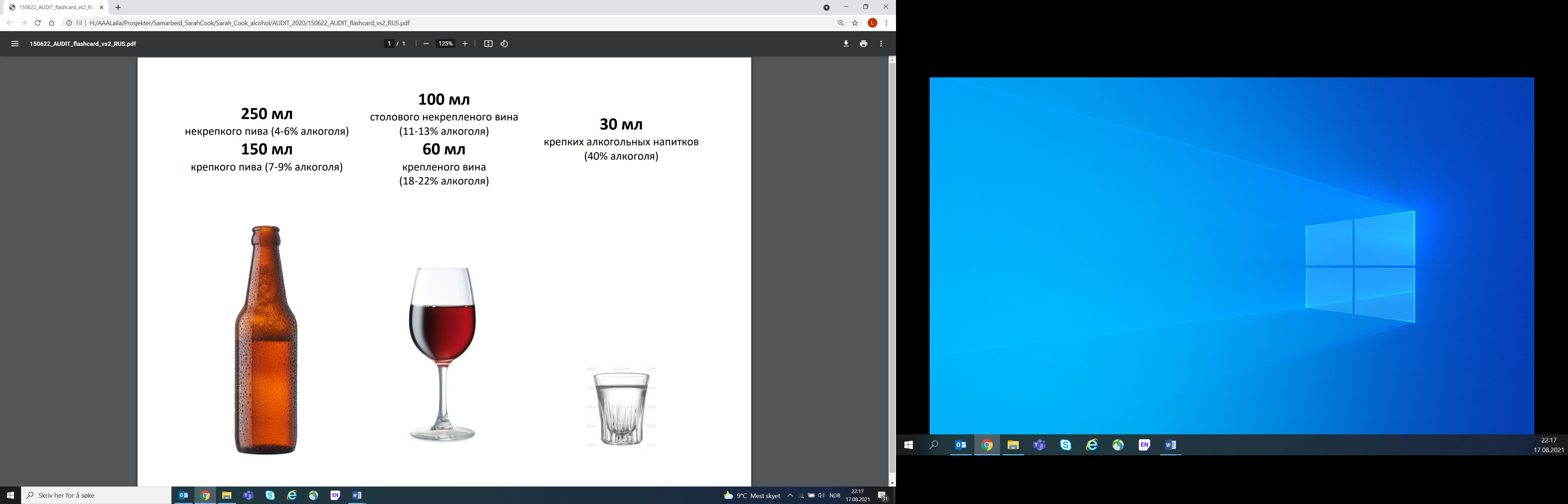

Supplement: sj-docx-3-sjp-10.1177_14034948211063656 – Supplemental material for Hazardous alcohol consumption and problem drinking in Norwegian and Russian women and men: The Tromsø Study 2015–2016 and the Know Your Heart study 2015–2018 [file sj-docx-3-sjp-10.1177_14034948211063656.docx]
